# Natural Killer Cell Cytokine Response to *M. bovis* BCG Is Associated with Inhibited Proliferation, Increased Apoptosis and Ultimate Depletion of NKp44^+^CD56^bright^ Cells

**DOI:** 10.1371/journal.pone.0068864

**Published:** 2013-07-15

**Authors:** Damien Portevin, Douglas Young

**Affiliations:** Division of Mycobacterial Research, MRC National Institute for Medical Research, Mill Hill, London, United Kingdom; Centre de Recherche Public de la Santé (CRP-Santé), Luxembourg

## Abstract

*Mycobacterium bovis* BCG, a live attenuated strain of *M. bovis* initially developed as a vaccine against tuberculosis, is also used as an adjuvant for immunotherapy of cancers and for treatment of parasitic infections. The underlying mechanisms are thought to rely on its immunomodulatory properties including the recruitment of natural killer (NK) cells. In that context, we aimed to study the impact of *M. bovis* BCG on NK cell functions. We looked at cytotoxicity, cytokine production, proliferation and cell survival of purified human NK cells following exposure to single live particles of mycobacteria. We found that *M. bovis* BCG mediates apoptosis of NK cells only in the context of IL-2 stimulation during which CD56^bright^ NK cells are releasing IFN-γ in response to mycobacteria. We found that the presence of mycobacteria prevented the IL-2 induced proliferation and surface expression of NKp44 receptor by the CD56^bright^ population. In summary, we observed that *M. bovis* BCG is modulating the functions of CD56^bright^ NK cells to drive this subset to produce IFN-γ before subsequent programmed cell death. Therefore, IFN-γ production by CD56^bright^ cells constitutes the main effector mechanism of NK cells that would contribute to the benefits observed for *M. bovis* BCG as an immunotherapeutic agent.

## Introduction

*Mycobacterium bovis* BCG (Bacillus Calmette–Guérin) has been widely used since 1921 and, despite variable protective levels, remains the only available vaccine against tuberculosis [1–3]. With 90% global coverage [4], the injection of live *M. bovis* BCG is safe, excepting circumstances of Mendelian or acquired immuno-deficiencies. This attenuated mycobacterial strain has also been assessed for the treatment of unrelated diseases with particular success against malignancies. Early observations suggested a lower incidence of cancers in TB patients, and in 1935 Holmgren used tuberculin and BCG to successfully prevent tumor progression [5]. Progressively, the use of live mycobacteria or their derivatives to treat cancer was overtaken by the advent of modern chemotherapy. However, the adjuvant properties of *M. bovis* BCG have recently been shown to contribute to the treatment success of first grade colon cancer [6] as well as parasitic infection such as diffuse cutaneous Leishmaniasis [7]. Most importantly, intra-vesical application of live *M. bovis* BCG is currently the recommended adjuvant treatment following surgical intervention of superficial bladder carcinomas [8,9]. The underlying mechanisms are not completely understood, but the recruitment of NK cells during mice and human *M. bovis* BCG infections has been described [7,10] and appeared to be essential for effective BCG immunotherapy in a murine bladder cancer model [11]. The observed correlation between preferential induction of a Th1 response and success of BCG immunotherapy suggests that production of IFN-γ makes a key contribution to positive disease outcome. In fact, Natural Killer (NK) cells provide the primary source of IFN-γ during cord blood exposure to *M. bovis* BCG [12]. Studies using NK cells isolated from adults showed that they can produce IFN-γ following direct contact with *M. bovis* BCG in the absence of accessory cells and that this is at least partially mediated by signalling through Toll-like receptor 2 [13,14]. Furthermore, NKp44, a receptor that is expressed by NK cells as well as γδ T cells can also bind mycobacteria [15]. Human NK cell populations are not uniform and include sub-populations that vary in their effector function. According to the expression of surface markers, CD56^bright^/CD16^-^ and CD56^dim^/CD16^+^ NK cells can be distinguished [16]. Expressing a different set of chemokine receptors, these two subsets are likely to traffic differently upon inflammation [17]. For instance, CD56^bright^/CD16^-^ NK cells were found preferentially enriched within tuberculous pleural fluid [18], and CD16^+^ NK cells were shown to make a potent contribution through perforin mediated cytotoxicity in a mouse model of BCG immunotherapy [19]. Furthermore, an HLA-DR expressing subset of human NK cells has been shown to react and expand following contact with *M. bovis* BCG and IL-2 [20].

To understand the fundamental adjuvant properties of *M. bovis* BCG, and to assist in their rational exploitation in combating disease, we have characterised the effect of *M. bovis* BCG on cytokine production, cytotoxic function, and cell fate of human NK cells in an *ex vivo* co-culture model. We describe a sequential programme involving IFN-γ production followed by apoptosis of a subset of CD56^bright^ NK cells.

## Results

### Effect of *M. bovis* BCG on cytokine response and cytotoxicity of human NK cells

**Figure 1:**
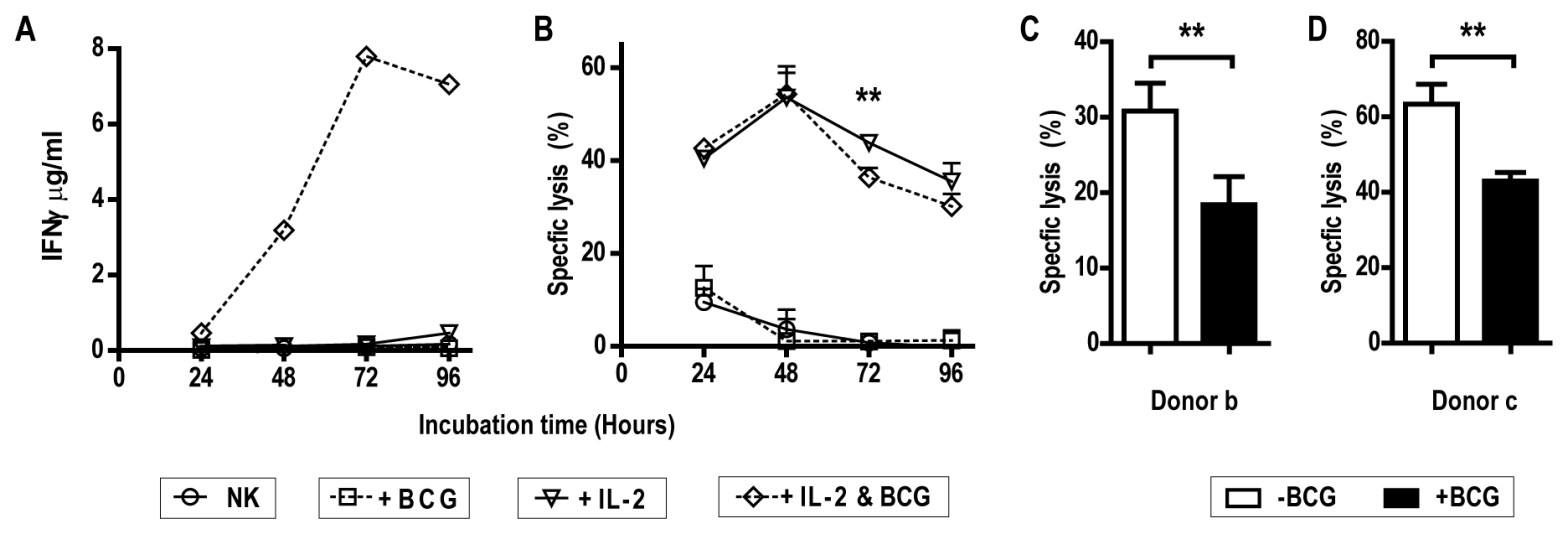
Prolonged exposure to *M. bovis* BCG affects NK cell cytoxicity. Human NK cells isolated from the blood of healthy donors were cultured in the presence or absence of interleukin-2 (IL-2) (100U/ml) and/or *M. bovis* BCG (MOI 1:4). A) Cell supernatants from a single donor were assayed for IFN-γ every 24h and for up to 96h post-culture. B) Recovered NK cells were assayed for cytotoxicity against the erythroleukemia line, K562, at an effector:target ratio of 1:1. C) and D) Bar graph showing significant reduction of NK cell cytotoxicity against K562 cells in the presence of IL-2 following 72h of exposure or not with mycobacteria from independent experiment and donor (Mean +/- SD of technical triplicates, unpaired t test, **p<0.01).

Given the potency of *M. bovis* BCG to prevent the recurrence of bladder carcinoma following surgical resection, we first aimed to evaluate the cytotoxic properties of human Natural Killer cells exposed or not to mycobacteria against a tumor cell line. NK cells isolated from peripheral blood of a healthy donor were cultured with single cell suspensions of live *M. bovis* BCG over a 96 hour time course. Every 24h, NK cells were recovered to measure their cytotoxicity against the MHC class I deficient K562 cell line. In parallel, we followed the production of IFN-gamma (IFN-γ) in the supernatant of NK cells with or without exposure to mycobacteria in order to assess the NK cell reactivity to the mycobacterial suspension. As shown previously [13], NK cells cultured with interleukin-2 (IL-2) alone produced only small amounts of interferon-gamma (IFN-γ), whereas combined stimulation with cytokine and *M. bovis* BCG resulted in progressive release of substantial amounts of IFN-γ up to 72h of co-culture (Figure 1A). In contrast, while NK cells that had been rested in complete medium in the absence of IL-2 displayed very little cytotoxicity against the MHC class I deficient K562 tumour cell line, addition of IL-2 alone enhanced NK cell cytotoxicity substantially from 24h up to 48h of culture (Figure 1B). During the first 48h of culture in the presence of IL-2, addition of *M. bovis* BCG did not affect the ability of human NK cells to lyse their target, though we observed a small but significant decrease in lytic activity over the subsequent 48h of incubation. The decrease in cytotoxicity against K562 cells observed after 72h mycobacterial exposure was consistent across independent experiments using NK cells from different blood donors (Figure 1C). Noteworthy, the reduced lysis of the K562 cell line coincided with the time when IFN-γ production reached a plateau.

### CD56^bright^ NK cells constitute the major source of IFN-γ in response to mycobacteria

**Figure 2:**
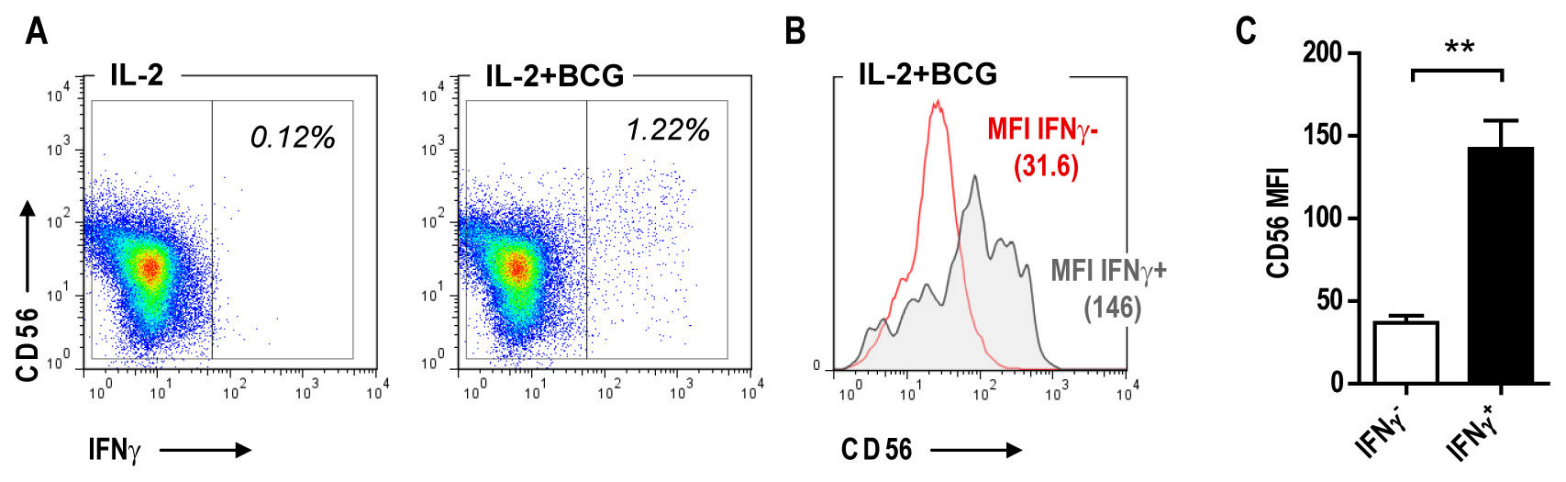
IFN-γ producing NK cells in response to mycobacteria express higher levels of CD56. Human NK cells were cultured in the presence of IL-2 (100U/ml) and/or *M. bovis* BCG (MOI 1:1) for a total of 24h including Brefeldin A treatment. A) FACS dot-plot showing the gating strategy to distinguish IFN-γ producing NK cells following BCG stimulation. B) CD56 overlay histogram comparing the expression of CD56 between IFN-γ producing and non-producing NK cells. C) Bar graph showing significant increase of CD56 mean fluorescence intensity (MFI) from IFN-γ producing NK cells following BCG stimulation across different donors (n=4, mean +/- SD, paired t test, **p<0.01).

**Figure 3:**
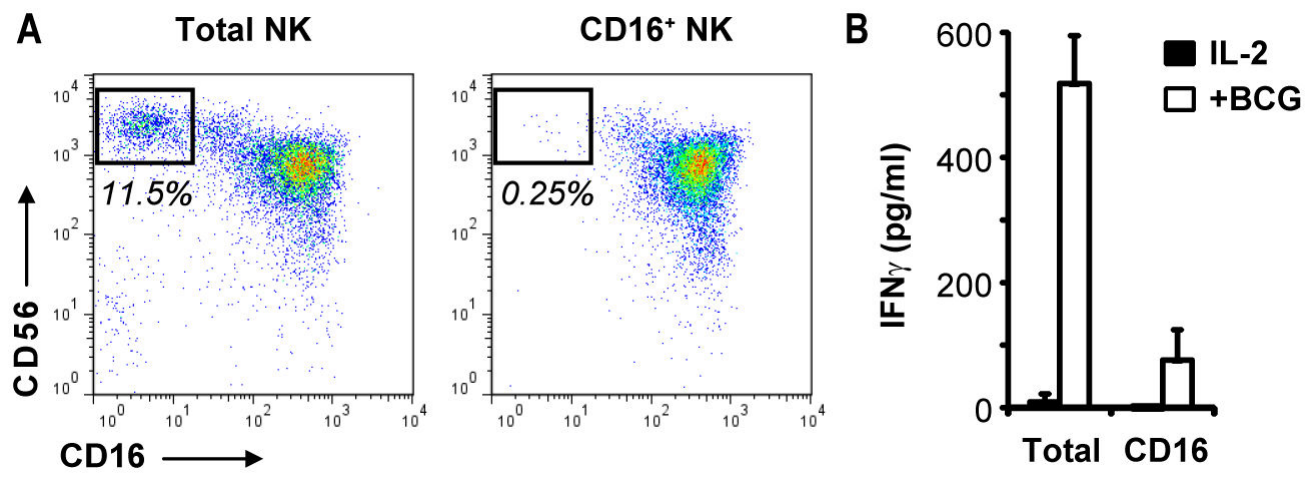
CD56^bright^ NK cell subset constitutes the main source of IFN-γ following mycobacterial exposure. A) CD56 versus CD16 FACS plot analysis of the total population of NK cells (left plot) isolated from the blood of a healthy human donor in comparison to CD16^+^ isolated NK cells from the same donor (right plot). B) Bar graph showing the production of IFN-γ after 72h of exposure with *M. bovis* BCG (MOI 1:4) from total NK cells in comparison to an equal number of autologous CD16^+^ NK cells (n=2, mean +/- SD of technical replicates).

To identify the source of IFN-γ within the population of NK cells during co-culture with IL-2 and *M. bovis* BCG, we measured *de novo* IFN-γ production using intracellular cytokine staining and flow cytometry after 24h of stimulation. Only a fraction of the NK cells produce IFN-γ in response to mycobacterial stimulation (Figure 2A). NK cells are usually stratified as dim or bright, according to the level of CD56 expression [16]. Across four independent donors, the level of CD56 expression was found to be significantly higher for IFN-γ positive cells, and so suggesting that CD56^bright^ cells represent the major source of IFN-γ (Figure 2B, 2C). CD56^bright^ NK cells differ notably from the CD56^dim^ subset by reduced expression of the low affinity Fc receptor, CD16. Consequently, selection of CD16^+^ cells results in a population of NK cells depleted for CD56^bright^ cells (Figure 3A). When exposed to *M. bovis* BCG and at the same cell density, the CD56^bright^-depleted cells released substantially less IFN-γ than the undepleted population, further demonstrating that the major part of IFN-γ secretion in response to mycobacteria originates from CD56^bright^ NK cell subset (Figure 3B).

### *M. bovis* BCG prevents IL-2 induced NKp44 expression and proliferation of CD56^bright^ NK cells

**Figure 4:**
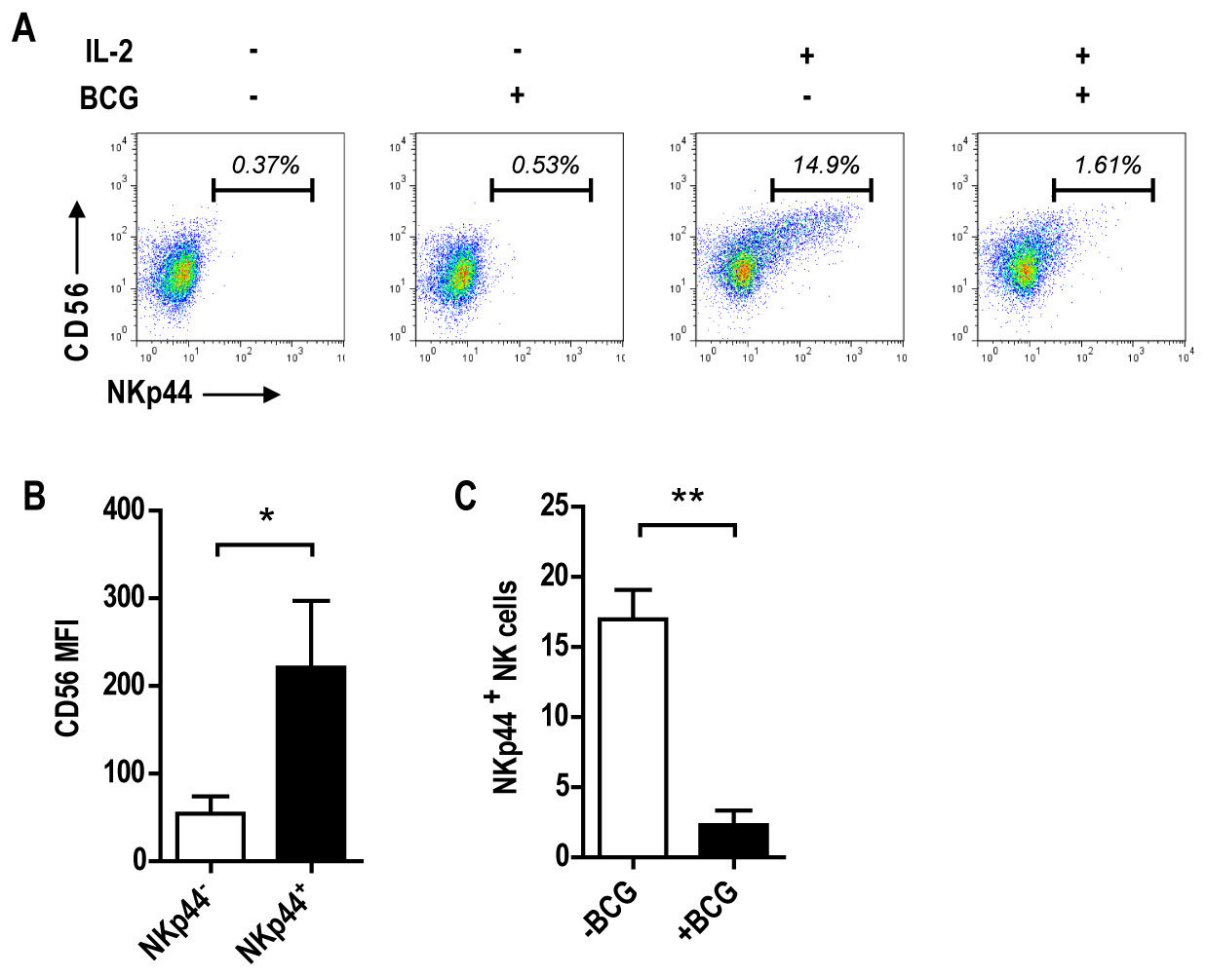
IL-2 induction of NKp44 expression on CD56^bright^ NK cells is inhibited by mycobacteria. A) FACS plot analysis of NKp44 expression from NK cells cultured in the presence or the absence of IL-2 (100U/ml) and or *M. bovis* BCG (MOI 1:5) for 5 days. B) Bar graphs showing B) significant increase of CD56 MFI of the NKp44^+^ NK cell population and C) significant decreased frequencies of IL-2 induced NKp44^+^ NK cell in the presence of mycobacteria from three independent experiments and donors (Paired t test, *p<0.05, **p<0.01).

Although exogenous IL-2 alone does not elicit IFN-γ production, it does have important effects on human NK cell populations. This notably includes induction of surface expression of NKp44 [21], a receptor described to bind mycobacteria [15]. IL2-induced expression of NKp44 is significantly associated with the CD56^bright^ NK cell subset (Figure 4B). In contrast to its stimulatory effect on IFN-γ production, the presence of *M. bovis* BCG substantially decreased the frequency of NKp44 positive NK cells and therefore CD56^bright^ NK cells (Figures 4A, and 4C).

**Figure 5:**
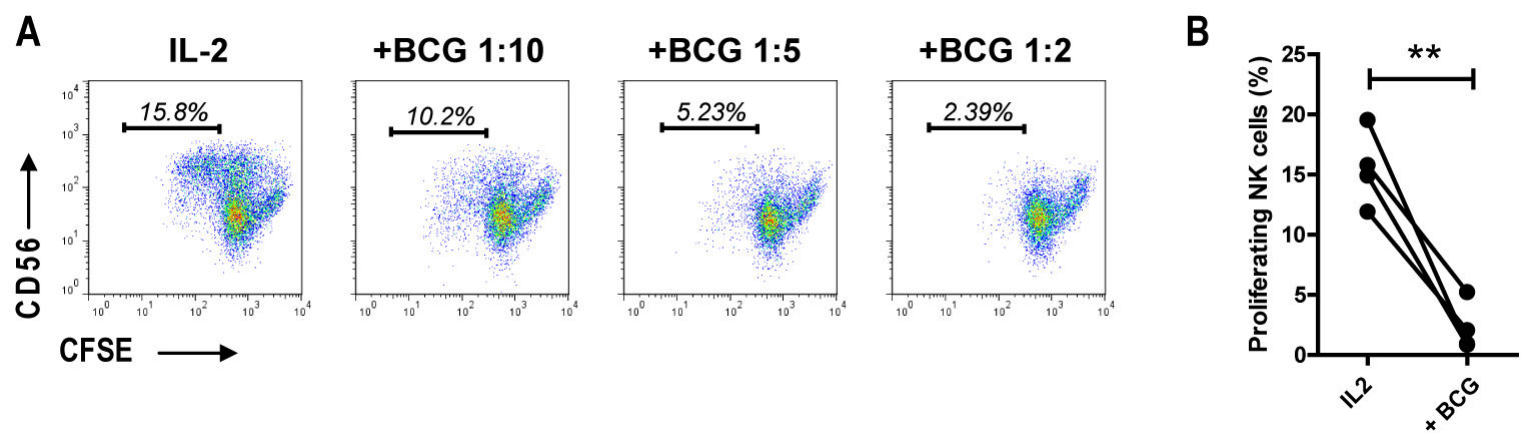
*M. bovis* BCG inhibits IL-2 induced proliferation of CD56^bright^ NK cells. A) Purified NK cells from a healthy human donor were labelled with CFSE and cultured for 7 days in the presence of IL-2 (100U/ml) +/- *M. bovis* BCG at various MOI before flow cytometry analysis. Dose dependent inhibition of NK cell proliferation by mycobacteria was observed. B) Joined dot plot illustrating the reproducibility of NK cell proliferation inhibition by mycobacteria across different NK cell preparation across independent donors (n=4, paired t test, **p<0.01).

IL-2 also promotes proliferation of NK cells allowing potential *in vitro* expansion [22]; in particular, CD56^bright^/CD16^-^ NK cells proliferate in response to a low dose (pM) of IL-2 [23]. Consistent with this, the fraction of NK cells identified by CFSE dilution as proliferating during culture for 7 days in the presence of IL-2 fall predominantly within the CD56^bright^ subset (Figure 5A). In parallel with the effect on NKp44 expression, addition of *M. bovis* BCG efficiently prevented IL-2-induced proliferation of CD56^bright^ NK cells in a dose dependent manner. Summarizing results from 3 independent experiments, we observed consistent inhibition of NK cell proliferation by mycobacteria using NK cell preparations from four different donors (Figure 5B).

### IL-2 dependent induction of NK cell apoptosis by *M. bovis* BCG

**Figure 6:**
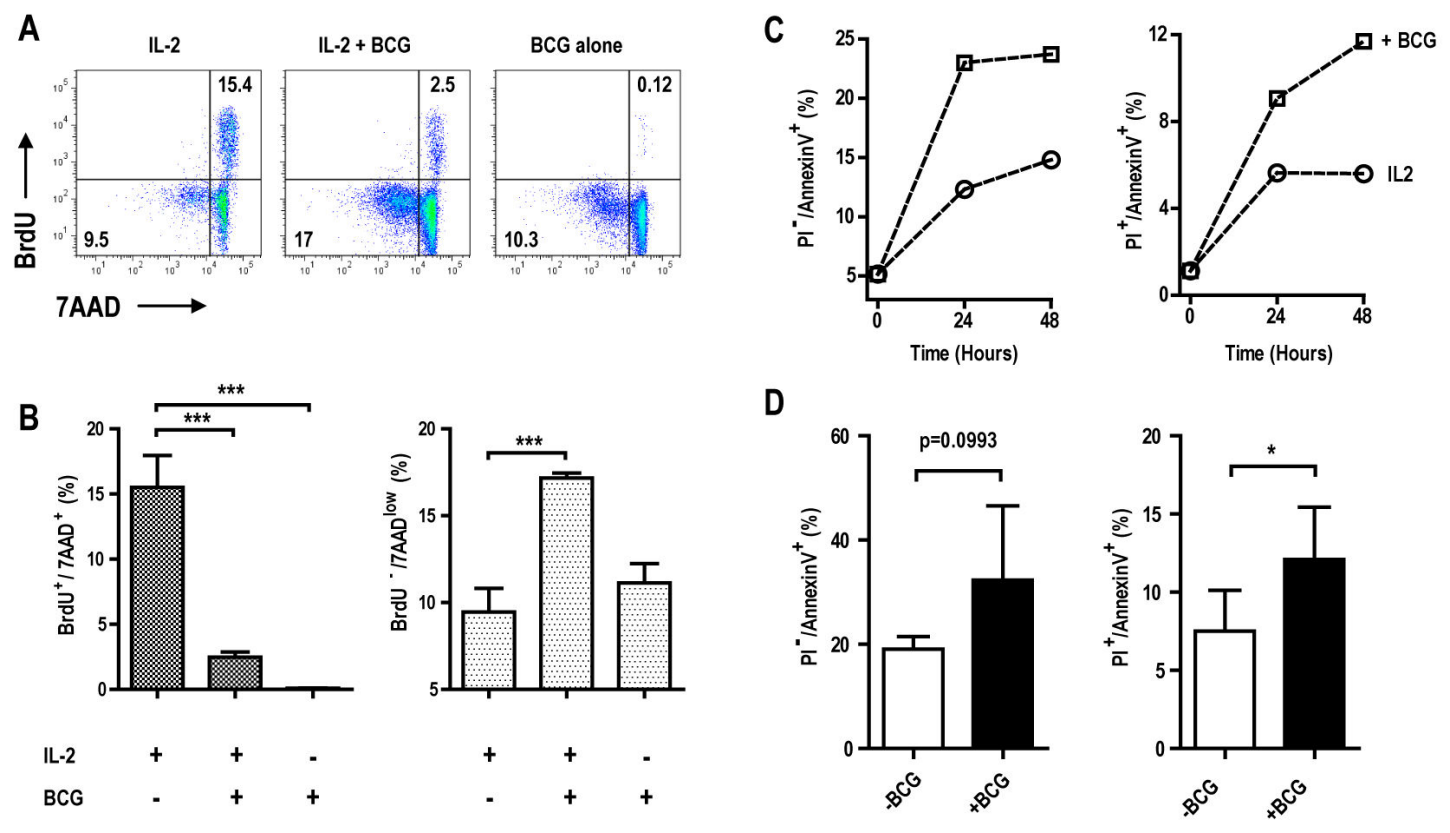
IL-2 dependent induction of NK cell apoptosis by *M. bovis* BCG. A) Representative FACS dot-plot of purified NK cells co-cultured or not with *M. bovis* BCG (MOI 1:5) and IL-2 (100U/ml) for 4 days before BrdU incorporation, antibody detection and 7AAD staining (One of three technical replicates). B) Bar graph showing on the left, the frequency of replicating events (BrdU^+^/7AAD^+^) and, on the right, the frequency of apoptotic events (BrdU^-^/7AAD^low^) of NK cells from one donor exposed or not to mycobacteria and IL-2 (Mean +/- SD of technical triplicates, unpaired t test, *** p<0.001). C) Frequencies of early (PI^+^/Annex V^-^) versus late apoptotic events (PI^+^/Annex V^+^) over the time of purified NK cells cultured or not with *M. bovis* BCG (MOI 1:1) and IL-2 (100U/ml). D) Bar graph summarizing frequencies of apoptotic events after 48h of culture with or without mycobacteria and IL-2 from independent experiments and donors (n=3, paired t test, *p<0.05).

To further characterise the fate of NK cells co-cultured in the presence of IL-2 and *M. bovis* BCG, we compared uptake of BrdU as a marker of proliferation with 7AAD staining as a marker of nuclear integrity. Consistent with measurement of CFSE dilution, *M. bovis* BCG significantly reduced the frequency of BrdU^+^ cells in comparison to that observed with IL-2 alone (Figure 6A). Furthermore, we observed a significant increase in cells detected in the BrdU^-^/7AAD^low^ gate, indicating enhanced chromosomal DNA fragmentation in cultures containing both IL-2 and *M. bovis* BCG (Figure 6B). *M. bovis* BCG on its own did not induce any significant increase of the frequency of such events when compared to IL-2 alone. To further test whether reduced 7AAD staining reflected programmed cell death, we performed Annexin-V binding and PI permeability assays on NK cells co-cultured with IL-2 in the presence or absence of mycobacteria. Early (AnnexinV^+^/PI^-^) and late (AnnexinV^+^/PI^+^) apoptotic events were markedly increased in the presence of mycobacteria. This effect was consistent across multiple NK cell preparations from multiple donors (Figure 6C, 6D).

## Discussion

NK cells are recruited to the site of mycobacterial infection in the context of pulmonary and extra-pulmonary tuberculosis [13,24] and also during experimental infection with *M. tuberculosis* and *M. bovis* BCG [10,25]. NK cells have pleiotropic functions overlapping to some extent the capacities of CD4 and CD8 T cells through their cytotoxic potential and their ability to produce various cytokines and chemokines [26]. With the aim of exploring the potential role of NK cells in protection and pathology during mycobacterial infection, and their contribution to the adjuvant properties of mycobacterial vaccines, we have characterised the impact of exposure to *M. bovis* BCG on the effector functions of human NK cells.

It is important to note that all the immunomodulatory properties of *M. bovis BCG* on NK cell cytotoxicity, proliferation and cytokine production summarized in this study are dependent on IL-2 co-stimulation and therefore NK cell activation that should be achieved *in vivo* following their extravasation to the site of infection. We observed that during the first 48h of contact when co-stimulated with IL-2, *M. bovis* BCG triggers the release of IFN-γ without affecting the cytotoxic activities of NK cells that are substantially enhanced by IL-2 itself. The release of cytokines by NK cells can happen concomitantly with the polarized secretion of perforin containing granules towards a target cell. However, the IFN-γ secretory pathway has been shown to be distinct from cytotoxic granules exocytosis, allowing the NK cell cytokine production to orchestrate the immune response independently of cytotoxicity [27]. Indeed, co-culture with *M. bovis* BCG and IL-2 did not enhance the natural cytotoxicity properties of human NK cells against the K562 leukaemia line although promoting the production of IFN-γ. On the contrary, prolonged exposure to mycobacteria resulted in a partial decrease of NK cell cytotoxicity. This is in contrast to a previous report describing enhanced cytotoxicity in the presence of *M. bovis* BCG [28]. This discrepancy might be due to the fact that we have used substantially lower effector: target ratios that are likely to miss small effects. Although not significantly potentiated by mycobacteria themselves, it is very likely that a significant contribution of NK cell in BCG immunotherapy of superficial bladder carcinoma or tuberculosis infection could originate from their cytotoxic arm once recruited at the site of infection.

In contrast to the limited effects on cytotoxic function, co-culture of purified NK cells with *M. bovis* BCG and IL-2 resulted in a significant increase in production of IFN-γ. IL-2 is known to synergise with TLR ligands to trigger IFN-γ production by NK cells [29]. In the absence of antigen-presenting cells, direct recognition of mycobacteria by NK cells has been suggested to occur at least partially through TLR2 [14]. Intracellular cytokine staining and cell depletion identified a subset of CD56^bright^/CD16^-^ cells as the predominant source of IFN-γ. Interestingly, NKp44 was described to bind specifically to mycobacteria [15]. Surface expression of NKp44 is notably induced by IL-2 [21] and we showed here that this mostly concerns CD56^bright^ NK cells and that the presence of mycobacteria significantly affected this induction. Further work is still needed to clarify whether mycobacterial signalling through TLR2 prevents NKp44 expression at the surface of CD56^bright^ NK cells and eventually their proliferation or if NKp44 binding of BCG itself is responsible for this inhibition and ultimately apoptosis induction and depletion of CD56^bright^ NK cells. In any case, CD56^bright^ NK cells seem to constitute the major protagonist of the NK cell response to *M. bovis* BCG.

The CD56 dim/bright dichotomy that normally distinguishes two subsets among resting NK cells according to their cytotoxic versus cytokine production propensities shows limitations when extended to *ex vivo* activated NK cells. Indeed, following experimental exposure with IL-2, up-regulation of the CD56 antigen by NK cells has been described [30]. Nevertheless, preferential expansion of the CD56^bright^ NK cell subset has also been observed following infusion of a low dose of IL-2 to patients with advanced cancers [31]. This suggests that a dim/bright transition can occur *in vivo*. However, despite a potential induction of CD56 expression upon IL-2 exposure, our depletion experiment suggests that the majority of mycobacteria induced secretion of IFN-γ originates from *bona fide* CD56^bright^ NK cells. Nevertheless, as observed by intracellular cytokine staining, CD56^dim^ NK cells also accumulated IFN-γ in contact with mycobacteria. This production could also contribute to the immune response to *M. bovis* BCG when released in a polarized fashion together with perforin containing-granules during further cytotoxic event.

Although a previous report described induction of NK cell proliferation following exposure to *M. bovis* BCG [32], we found that mycobacteria alone had no effect on proliferation, but rather caused a pronounced inhibition of IL-2 induced proliferation. The reduced proliferative response was associated with enhanced apoptosis in cells co-cultured with mycobacteria in the presence of IL-2. A limitation of our study resides in the fact that the number of donors within individual approaches is relatively low. However, our observations are supported by different read-outs with converging conclusions (CFSE vs. BrdU and 7AAD vs. AnnexinV). Furthermore, this effect is reminiscent of a previous report showing that a combination of cytokines sufficient to stimulate the production of IFN-γ by human NK cells also triggered NK cell apoptosis [33]. As suggested by these authors, this programme provides a mechanism to limit the inflammatory response in order to prevent excessive inflammation. It can be anticipated that apoptosis of activated cells will further limit the short half-life of human NK cells, reducing the likelihood of an NK cell memory compartment comparable to that described in mice in the case of CMV infection [34]. Interestingly, NK cell apoptosis was also observed in the case of tuberculosis pleurisy highlighting further their apoptotic tendency *in vivo* during the natural course of mycobacterial infection [35].

In summary, while we do not exclude a possible contribution of their cytotoxic arm, we propose that a major effector function of NK cells in BCG immunotherapy of superficial bladder carcinoma or tuberculosis infection involves the production of IFN-γ by a subset of CD56^bright^ cells. NK cells driven into a terminally differentiated effector state subsequently undergo programmed cell death as part of a self-limiting response.

## Methods

### Blood samples, cells and cell cultures

Fresh blood packs (Buffy coats) from healthy adult donors were purchased anonymously from National Blood Services, London, UK. Ethical approval and informed consent were obtained prior to blood donation according to the Guidelines for the Blood Transfusion Services in the United Kingdom, 7th Edition 2005; for details and updates: UK Blood Transfusion & Tissue Transplantation Services Website. Available: http://www.transfusionguidelines.org.uk/. Accessed 2013 June 11. Peripheral blood mononuclear cells (PBMCs) were prepared on a Ficoll-Paque density gradient (Amersham Biosciences AB, Uppsala, Sweden) by centrifugation (800 g, 30 min at room temperature), washed twice and frozen in RPMI 1640-FCS(5%)-DMSO (8.7%)-methyl-cellulose(0.1%). Viability after recovery was systematically checked and above 95%. A 2h resting period was respected before initiating experiments. NK cells were selected from PBMCs using NK isolation kit II, CD16^+^ NK cell selection was performed using CD56^+^CD16^+^ NK Cell Isolation Kit according to manufacturer’s recommendations. Cell purity checked by flow cytometry was always >95%. Cells were cultured in complete RPMI 1640 medium, including 1 mM sodium pyruvate, and 1% heat-inactivated foetal calf serum in flat-bottom tissue culture treated 96 well plates at a density of 7.5.10^5^ cells/cm^2^. Recombinant IL-2 was purchased from PeproTech EC Ltd. K562 cell line was obtained from European Collection of Cell Cultures and cultured in 2500 mm^2^ flasks kept vertical. Cells were counted every 48h and cell density adjusted to 3.10^6^ cells in 10ml of RPMI 1640-glutamine (2mM), sodium pyruvate (1mM) and FCS (10%).

### BrdU incorporation and apoptosis analysis

Freshly isolated NK cells were cultured for 4 days with IL-2 (400u/ml) and/or *M. bovis* BCG (1:5). Bromodeoxyuridine (BrdU) incorporation was initiated for 16 hours before processing the cells using FITC/BrdU FlowKit (BD PharmingenTM) following manufacturer’s recommendations. Apoptosis experiments were performed using FITC Annexin V Apoptosis Detection Kit I from BD Pharmingen™ following manufacturer’s recommendations.

### CFSE dilution analysis

For CFSE cellular labelling, a 10mM stock solution of CFDA-SE (Invitrogen) in DMSO was freshly diluted in PBS (1/50000, V/V) and immediately used to resuspend cells at 5.10^6^ cells/ml and incubated for 8 min at 37°C. The reaction was stopped by adding one volume of FCS and cells were washed twice with PBS before culture.

### Culture and preparation of mycobacterial suspension

*M. bovis* BCG Pasteur was grown at 37°C in Middlebrook 7H9 broth supplemented with ADC (Becton Dickinson, Co, Sparks, USA) to mid-exponential growth phase and pelleted at room temperature. Single cell bacterial suspension was prepared as previously described (N’Diaye et al., 1998). Briefly, the medium was discarded, bacteria were dispersed by shaking for 1 minute with glass beads (3 mm diameter), and resuspended in PBS, pH 7.4. The remaining clumps were removed by centrifuging the supernatant for 10 minutes at 200g. In order to establish precise bacterial counts before and after freezing aliquots with glycerol (5% final V/V) and storage at -80°C, bacterial suspension were systematically plated on Middlebrook 7H11-agar plates supplemented with OADC (Becton Dickinson, Co, Sparks, USA) and plates incubated at 37°C for 14 days before reading.

### Cytokine production analysis

Cell free culture supernatants were filtered using 0.2µm 96-well filter plates (Corning) before detection of IFN-γ using either ELISA kit (Peprotech Ltd).

### Flow cytometry reagents and analysis

Anti-CD3-FITC (clone UCHT1) and anti-CD16-PE (clone 3G8) were purchased from Beckman Coulter, anti-NKp44-PE (clone 2.29) from Miltenyi Biotec, anti-CD56-PE (clone B159), anti-CD56-PE-Cy7 (clone B159) and anti IFN-γ-PE-C7 (clone 4S.B3) from BD Biosciences. Brefeldin A (final concentration 10µg/ml) was added 6 hours before antibody staining. Fixation and permeabilization was performed using BD cytofix/cytoperm kit from BD biosciences. Cells were analysed on a BD Biosciences FACSCalibur flow cytometer and data processed using FlowJo 7.6.4.

### Cytotoxicity assay

K562 target cells were loaded in RPMI containing calcein AM (Invitrogen) at 10µg/ml for 30 minutes and washed before incubation with 10^5^ NK cells (E:T ratio 1:1) in U-shaped plates with complete RPMI medium for 4h. Released fluorescence was measured with an excitation filter set at 485 nm and emission filter at 520 nm on a Polarstar Galaxy plate reader (BMG Labtechnologies, Germany). Percent of specific lysis was defined as (Experimental release (ER) – Spontaneous release (SR)) / (Maximal release (MR) – SR) *100, where ER represents the signal in the presence of effectors cells, SR the signal in the absence of effectors cells and MR the signal after lysis with Triton X100 (1% final). Experiments were performed on independent triplicates or more.

### Graphics and statistical analysis

Graphs and statistical analysis were performed using GraphPad Prism 5 software. Unless the direction of the association was expected prior to performing the assays, two-tailed statistical test were systematically performed.
